# iPSC-derived NK cells engineered with CD226 effectively control acute myeloid leukemia

**DOI:** 10.1186/s40164-025-00686-9

**Published:** 2025-07-07

**Authors:** Runze Cai, Binyan Lu, Xiangyu Zhao, Shixin Zhou, Yang Li

**Affiliations:** 1 Department of Cell Biology, School of Basic Medical Sciences, Peking University Stem Cell Research Center, Peking University, Beijing, China; 2 Guangzhou Regenverse Therapeutics Co.,Ltd, Guangzhou, China; 3 Peking University Institute of Hematology, Peking University People’s Hospital, Beijing, China

**Keywords:** Acute myeloid leukemia, iPSC-derived NK cells, CD226, Immunotherapy

## Abstract

CD226 plays a vital role in NK cell cytotoxicity, interacting with its ligands on tumor targets. Acute myeloid leukemia (AML) cells have developed mechanisms to escape NK cell cytotoxicity, including inducing downregulation of CD226 on NK cells. Induced pluripotent stem cell -derived NK (iPSC-NK) cells offer an important source of standardized off-the-shelf NK cell therapy to treat AML patients. In this study, we engineered iPSC-NK cells with CD226 to assess the ability of killing AML cells. iPSC-NK cells engineered with CD226 have a typical NK cell phenotype and demonstrate improved anti-AML activity and multiple cytokines releasing at low effector-to-target ratios. Transcriptomic analysis revealed upregulation of immune effector function pathways associated with cytotoxicity and immune activation in CD226-overexpression iPSC-NK cells. In an AML xenograft model, mice treated with CD226 overexpression iPSC-NK cells exhibited significantly reduced leukemia burden, prolonged survival, decreased systemic inflammation compared to those treated with Control iPSC-NK cells. Overall, our study provided evidence that iPSC derived-NK cells engineered with CD226 represent a promising candidate for off-the-shelf immunotherapy, particularly in AML and other CD226 ligand-expressing malignancies.

**Supplementary Information:**

The online version contains supplementary material available at 10.1186/s40164-025-00686-9.

## To the editor

NK cell-based immunotherapies have been shown to possess potent anti- AML effects without eliciting serious adverse effects [1]. CD226, an activating receptor expressed on NK cells, recognizes and binds to two ligands (CD122 & CD155) expressed on abnormal cells, thereby activating NK cells [2, 3]. In 2024, Kaito et al. reported that NK-92 cell lines modified with CD226 exhibited enhanced cytotoxic activity against AML cell lines via overcoming the inhibitory activity of TIGIT [4]. However, for the concern of tumorigenicity, NK-92 cells need to be irradiated prior to infusion to prevent uncontrolled proliferation, which limit the antitumor activity of these cells [5–8]. Induced pluripotent stem cells (iPSCs) can be precisely genetically modified and then efficiently differentiated to produce NK cells (iPSC-NK cells) [9, 10]. Therefore, iPSCs provide an important platform to produce standardized, off-the-shelf NK cells with improved anti-tumor activity [11]. Here, we overexpressed CD226 (OE226) using the PiggyBac transposon system in iPSC-derived NK (iNK) cells to evaluate the ability of killing AML cells in vitro and in vivo.

**Fig. 1:**
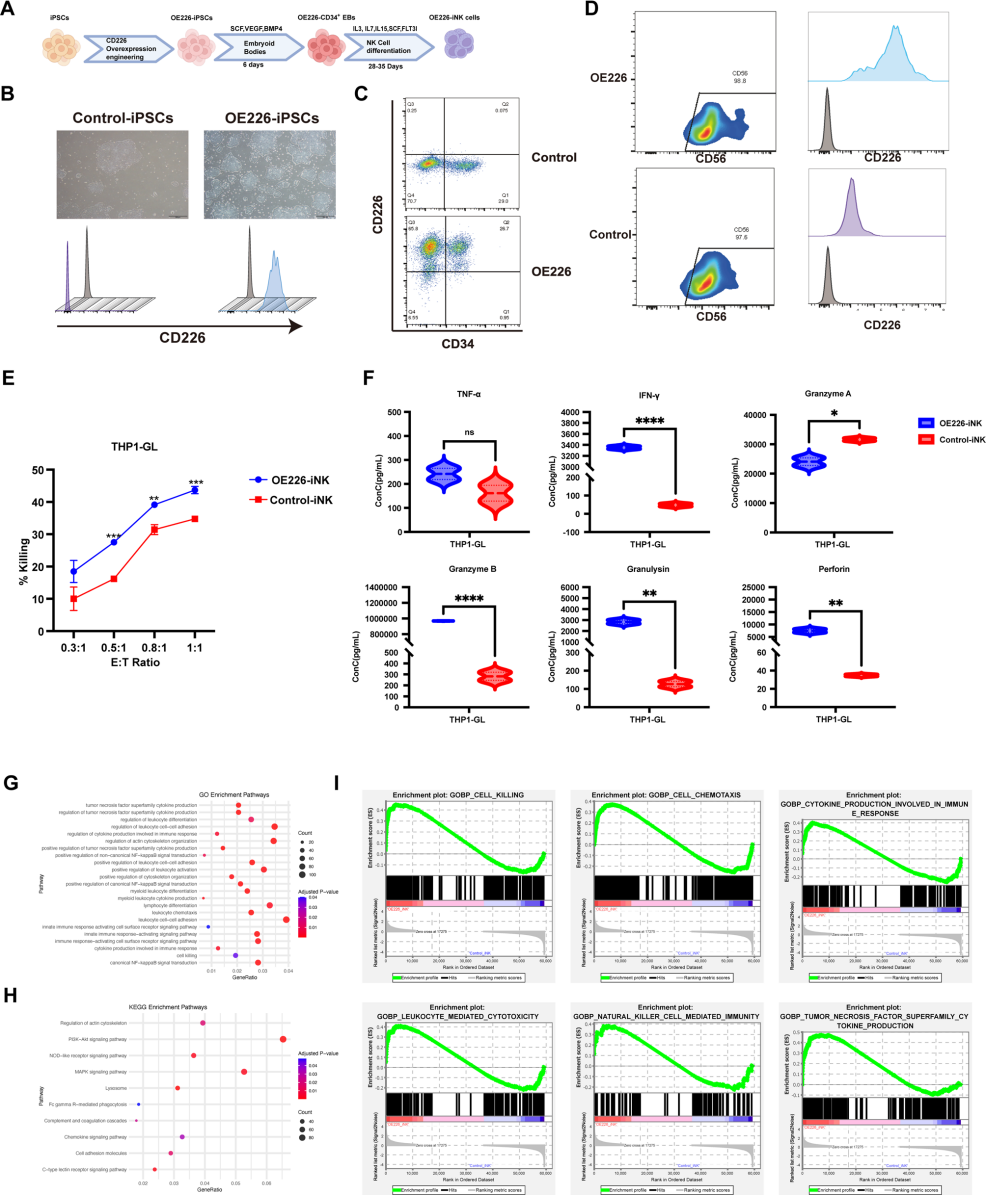
CD226-overexpressing iPSC-derived NK cells exhibit enhanced cytotoxicity and immune activation. **A** Schematic of the differentiation process from iPSCs to NK cells. **B** Morphology and flow cytometry analysis for CD226 expression of control and OE226-iPSCs. Scale bar, 100 μm. **C** Flow cytometry analysis for CD34 and CD226 expression in day 6 EB-derived cells. **D** Flow cytometry analysis for CD56 and CD226 expression in OE226-iNK (up) and control-iNK cells (down). Grey histograms represent isotype controls. **E** In vitro cytotoxicity assays against THP1-GL (AML) tumor cells at various effector-to-target (E: T) ratios (*n* = 3). Statistical significance was determined using unpaired student’s t-test. Statistical analysis: *p < 0.05; ** *p* < 0.01; **** *p* < 0.0001; ns, not significant. **F** Quantification of cytokines and cytotoxic molecules released by OE226-iNK and control-iNK cells after 24-hour co-culture with THP1 tumor cells at an E: T ratio of 1:1, analyzed using a bead-based multiplex cytokine assay and flow cytometry. Violin plots show secretion levels of TNF-α, IFN-γ, granzyme A, granzyme B, granulysin, and perforin. Data represent pooled technical replicates from three independent experiments (*n* = 3), Data are presented as mean ± SEM (*n* = 3); *p < 0.05; ** *p* < 0.01; **** *p* < 0.0001; ns, not significant (unpaired t-test). **G** Upregulated GO enrichment analysis of differentially expressed genes between OE226-iNK and control-iNK cells. **H** Bubble plot showing immune-related pathways enriched in the significantly upregulated genes. **I** GSEA (OE226-iNK vs. Control-iNK) showed enriched immune effector programs

The schematic of NK cell differentiation from iPSCs is summarized in Fig. 1A, following previously described protocol [12]. iPSCs engineered with CD226 (OE226-iPSCs) maintained typical undifferentiated morphology, expressing high levels of CD226 (Fig. 1B). OE226 iPSCs could form normal embryoid bodies (Fig. S1A), and successfully differentiated into CD34⁺ CD226^+^ hemogenic endothelium (Fig. 1C). Following 4 weeks of NK cell differentiation, iNK cells derived from both OE226 and Control iPSCs were generally morphologically indistinguishable (Fig. S1B), and expressed typical NK cell markers, including CD56, CD16, NKp44, NKp46, NKG2D, NKG2C, TRAIL, FasL, and the inhibitory receptor KIR2DL, supporting successful differentiation into NK cells (Fig. 1D, Fig. S2). More importantly, CD226 overexpression was further identified in OE226-iNK cells (Fig. 1D). OE226-iNK cells exhibited enhanced cytotoxicity against CD226 ligand positive THP1-GL (Fig. 1E, Fig. S3). We further evaluated cytokine secretion following co-culture with tumor targets. Violin plot analysis showed significantly increased secretion of IFN-γ, Granzyme B, Granulysin and Perforin in OE226-iNK cells (Fig. 1F).

We then performed transcriptomic analysis to compare overall gene expression diferences between OE226-iNK cells and Control-iNK cells. GO enrichment analysis revealed that differentially expressed genes were significantly enriched in immune-related biological processes, including TNF superfamily cytokine production, cell killing and innate immune activation (Fig. 1G). KEGG pathway analysis further supported these observations, showing upregulation of signaling pathways related to PI3K-Akt, MAPK, and NOD-like receptor signaling (Fig. 1H). GSEA demonstrated consistent upregulation of multiple immune effector pathways in OE226-iNK cells. Specifically, gene sets involved in cell killing, cytokine production, and natural killer cell mediated immunity were significantly enriched (Fig. 1I). These results were in agreement with enhanced NK function observed in cytotoxicity and cytokine release assays.

**Fig. 2:**
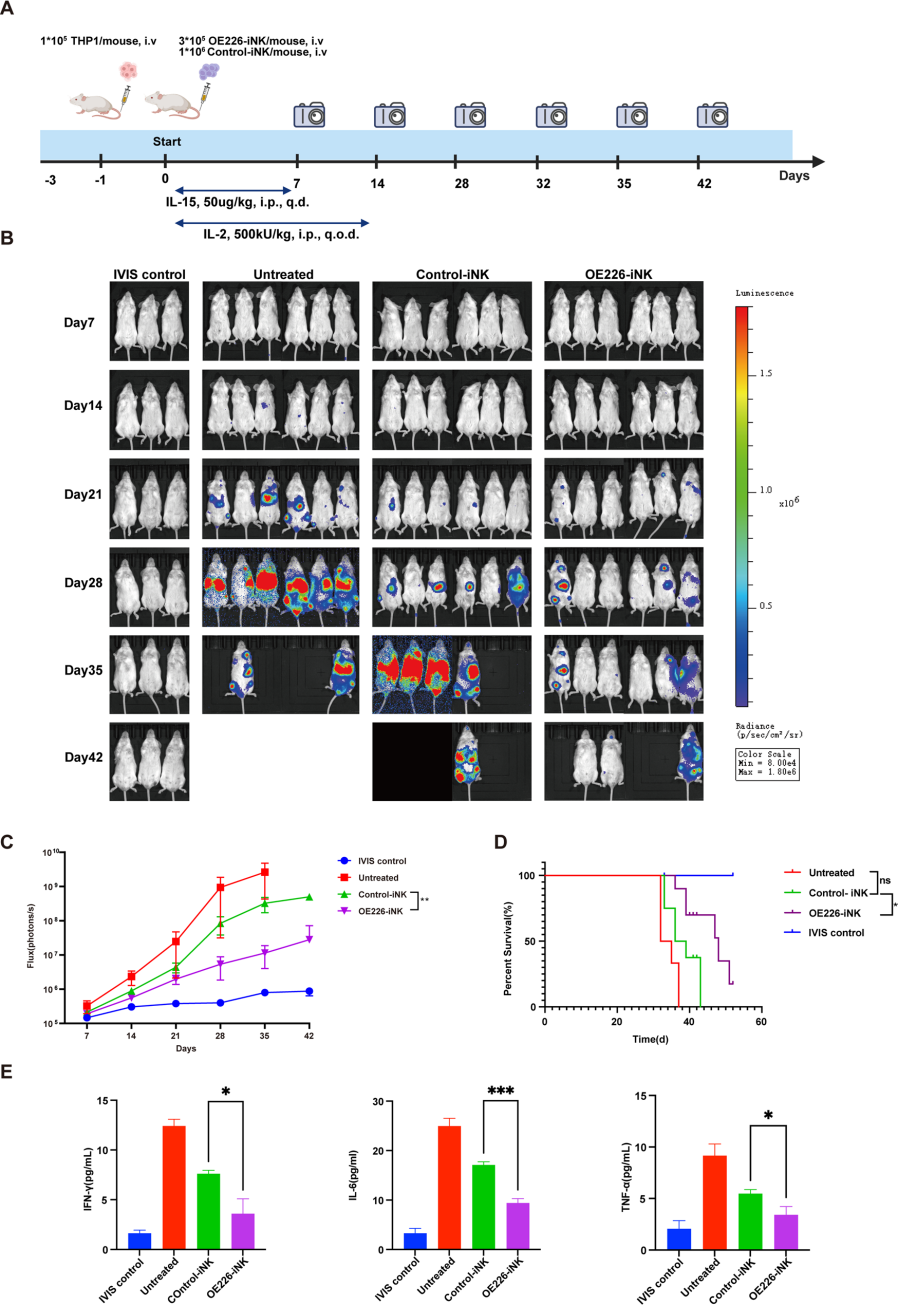
CD226-overexpressing iNK cells suppress AML progression and prolong survival in vivo. **A** Schematic diagram of the AML xenograft model and treatment timeline. NPG mice were injected intravenously with 1 × 10⁵ THP1-GL cells on day − 1. On day 0, mice received either 1 × 10⁶ control-iNK cells or 3 × 10⁵ OE226-iNK cells via tail vein injection. Recombinant human IL-15 (50 µg/kg/day) and IL-2 (500,000 U/kg/day) were administered intraperitoneally throughout the study. Created with BioRender. **B** Representative bioluminescence imaging (BLI) of tumor burden over time in four experimental groups: IVIS control, untreated, control-iNK, and OE226-iNK. imaging was performed weekly from day 7 to day 42 post-injection. **C** Quantification of tumor burden over time by BLI (mean ± SEM). Statistical significance was determined by two-tailed student’s t-test; **p < 0.01. **D** Kaplan–Meier survival analysis (*n* = 6 per group). *p < 0.05. **E** Serum levels of murine inflammatory cytokines (IFN-γ, IL-6, and TNF-α) were measured by ELISA at endpoint. Data are presented as mean ± SEM (*n* = 3); *p < 0.05, ***p < 0.001 (unpaired t-test)

We established a systemic AML xenograft model by intravenously injecting 1 × 10⁵ THP1-GL leukemia cells into immunodeficient NPG mice. At 24 h post-injection, mice were treated with either 3 × 10⁵ OE226-iNK cells or 1 × 10⁶ Control-iNK cells via intravenous injection. Tumor progression was monitored weekly using bioluminescence imaging (BLI) for six weeks. (Fig. 2A-B). BLI quantification revealed progressive leukemia development in the untreated group, with a steady increase in tumor burden over time. In contrast, OE226-iNK treated mice exhibited significantly reduced leukemia burden relative to both the Control-iNK treated and untreated groups (Fig. 2C). Kaplan–Meier survival analysis further demonstrated that OE226-iNK treatment significantly prolonged survival in AML-bearing mice. The median survival was 48 days in the OE226-iNK group, compared to 37.5 days in the Control-iNK group and 33.5 days in the untreated group (Fig. 2D). We also measured serum levels of murine inflammatory cytokines in AML-bearing mice. Compared to Control-iNK cell treatment, OE226-iNK-treated mice exhibited significantly lower concentrations of IL-6, IFN-γ, and TNF-α, indicating that CD226 overexpression may reduce systemic inflammation in the AML microenvironment (Fig. 2E).

Our study also has some limitations. Firstly, tumors used for xenograft models are cell line derived, while patient-derived xenograft model might be closer to the clinical scenarios. Secondly, therapeutic efficacy of various doses of OE226-iNK cells should be assessed in AML xenograft model. Thirdly, the precise ligand-specific mechanism of CD226-mediated cytotoxicity remains to be explored. Although our study has several limitations, it provides evidence for CD226 as a rational engineering target to develop potent iPSC-derived NK cell therapies against AML.

## Electronic supplementary material

Below is the link to the electronic supplementary material.

Supplementary Material 1

## Data Availability

https://www.ncbi.nlm.nih.gov/geo/query/acc.cgi?acc=GSE292274 Raw RNA-seq data performed during this study can be obtained from GSE292274.

## Abbreviations

AML: Acute myeloid leukemia
iPSC: Induced pluripotent stem cell
NK: Natural killer cell
iNK: iPSCs-derived NK
TIGIT: T cell immunoreceptor with Ig and ITIM domains
EB: Embryoid body
NPG: NOD. Cg-Prkdcscid Il2rgtm1Vst/Vst
IVIS: In vivo imaging system
E:T: Effector-to-target ratio
GZMB: Granzyme B
GO: Gene ontology
KEGG: Kyoto encyclopedia of genes and genomes
GSEA: Gene set enrichment analysis
BLI: Bioluminescence imaging
Fas-L: Fas ligand
IFN-γ: Interferon-γ
TNF-α: Tumor necrosis factor-alpha
TRAIL: TNF-related apoptosis-inducing ligand
SEM: Standard error of the mean

## References

[1] Huang R, Wang X, Yan H, Tan X, Ma Y, Wang M, et al. Safety and efficacy of CD33-targeted CAR-NK cell therapy for relapsed/refractory AML: preclinical evaluation and phase I trial. Exp Hematol Oncol. 2025;14(1):1. 39748428 10.1186/s40164-024-00592-6 PMC11694373

[2] Shibuya A, Shibuya K. DNAM-1 versus TIGIT: competitive roles in tumor immunity and inflammatory responses. Int Immunol. 2021;33(12):687–92. 34694361 10.1093/intimm/dxab085

[3] Mendelsohn CL, Wimmer E, Racaniello VR. Cellular receptor for poliovirus: molecular cloning, nucleotide sequence, and expression of a new member of the immunoglobulin superfamily. Cell. 1989;56(5):855–65. 2538245 10.1016/0092-8674(89)90690-9

[4] Kaito Y, Sugimoto E, Nakamura F, Tsukune Y, Sasaki M, Yui S, et al. Immune checkpoint molecule DNAM-1/CD112 axis is a novel target for natural killer-cell therapy in acute myeloid leukemia. Haematologica. 2024;109(4):1107–20. 37731380 10.3324/haematol.2023.282915 PMC10985452

[5] Handgretinger R, Lang P, André MC. Exploitation of natural killer cells for the treatment of acute leukemia. Blood. 2016;127(26):3341–9. 27207791 10.1182/blood-2015-12-629055

[6] Bachanova V, Cooley S, Defor TE, Verneris MR, Zhang B, McKenna DH, et al. Clearance of acute myeloid leukemia by haploidentical natural killer cells is improved using IL-2 diphtheria toxin fusion protein. Blood. 2014;123(25):3855–63. 24719405 10.1182/blood-2013-10-532531 PMC4064329

[7] Zhu H, Kaufman DS. Engineered human pluripotent stem cell-derived natural killer cells: the next frontier for cancer immunotherapy. Blood Sci (Baltimore Md). 2019;1(1):4–11. 10.1097/BS9.0000000000000023 PMC8974906 35402797

[8] Yao P, Liu YG, Huang G, Hao L, Wang R. The development and application of chimeric antigen receptor natural killer (CAR-NK) cells for cancer therapy: current state, challenges and emerging therapeutic advances. Experimental Hematol Oncol. 2024;13(1):118. 10.1186/s40164-024-00583-7 PMC11616395 39633491

[9] Li Y, Hermanson DL, Moriarity BS, Kaufman DS. Human iPSC-Derived natural killer cells engineered with chimeric antigen receptors enhance Anti-tumor activity. Cell Stem Cell. 2018;23(2):181–92. 30082067 10.1016/j.stem.2018.06.002 PMC6084450

[10] Zhu H, Blum RH, Bernareggi D, Ask EH, Wu Z, Hoel HJ, et al. Metabolic reprograming via deletion of CISH in human iPSC-Derived NK cells promotes in vivo persistence and enhances anti-tumor activity. Cell Stem Cell. 2020;27(2):224–37. 32531207 10.1016/j.stem.2020.05.008 PMC7415618

[11] Meng F, Zhang S, Xie J, Zhou Y, Wu Q, Lu B, et al. Leveraging CD16 fusion receptors to remodel the immune response for enhancing anti-tumor immunotherapy in iPSC-derived NK cells. J Hematol Oncol. 2023;16(1):62. 37316891 10.1186/s13045-023-01455-z PMC10265820

[12] Zhu H, Kaufman DS. An improved method to produce clinical-scale natural killer cells from human pluripotent stem cells. Methods Mol Biol. 2019;2048:107–19. 31396935 10.1007/978-1-4939-9728-2_12

