# Supplementary material for: iPSC-derived NK cells engineered with CD226 effectively control acute myeloid leukemia
Source: Exp Hematol Oncol. 2025 Jul 7;14:93. doi: 10.1186/s40164-025-00686-9 (PMC12232852; doi:10.1186/s40164-025-00686-9)
Supplement: Supplementary file 1 — Supplementary Material 1 [file 40164_2025_686_MOESM1_ESM.docx]

**CONTENT**

**Methods**

**Supplemental Figures**

Supplementary Figure 1. NK cell differentiation from Control and OE226-iPSCs.

Supplementary Figure 2. Flow cytometry analysis for surface immunophenotyping of OE226-iNK and Control-iNK cells.

Supplementary Figure 3. Flow cytometry analysis for CD226 ligand expression in THP1-GL cells.

**Supplemental Tables**

Supplementary Table 1. Antibodies used in this study.

**Abbreviations**

**Methods**

**Cell Lines and Culture Conditions**

THP1 cells were kindly provided by Dr. Xiangyu Zhao (Peking University People’s Hospital). Human iPSCs used in this study were previously established and characterized by our laboratory. iPSCs were maintained on Matrigel-coated plates (Corning) in mTeSR1 medium (STEMCELL Technologies) and passaged every 3–4 days using 0.5 mM EDTA (Thermo Fisher) according to the manufacturer’s instructions. THP1 cells were further engineered to stably express firefly luciferase and green fluorescent protein (GFP). THP1 cells were cultured in RPMI 1640 medium (Gibco) supplemented with 10% fetal bovine serum (FBS; Viva Cell) and 1% penicillin–streptomycin(Gibco).

**Plasmid Construction and Gene Cloning**

The PiggyBac transposon vector pPB-CG.EGFP-puΔtk was used as the backbone for gene integrations. The EGFP cassette was excised using NheI and XhoI restriction enzymes and the resistant puromycin cassette was reserved for stable expression screening. The DNA encoding human CD226 (NM_006566.4) was synthesized by Ruibiotech Co., LTD. (Beijing, China). The synthesized gene was digested with NheI and XhoI enzymes and inserting into the prepared the vector via T4 DNA ligation. All inserted constructs were verified by Sanger sequencing. The expression of CD226 was under the control of the CAG promoter.

**Generation of CD226 overexpression iPSCs**

For iPSC engineering, human iPSCs were electroporated with the CD226 construct (1 μg) and PiggyBac transposase helper plasmid (1 μg) using the Lonza 4D-Nucleofector X Unit and P3 Primary Cell 4D-Nucleofector Kit (LONZA, Cambridge, MA) with program CA-137. Following nucleofection, cells were selected with puromycin to obtain stable CD226-Overexpression (OE226) iPSC clones. CD226 expression was confirmed by flow cytometry, and positive clones were expanded and banked for downstream differentiation. iPSCs electroporated with an empty PiggyBac vector were selected with puromycin, serving as the control to CD226 overexpression.

**NK cell derivation and expansion from iPSCs**

Human iPSCs were differentiated into hematopoietic stem/progenitor cells and subsequently into NK cells as previously described protocol. Briefly, hiPSCs were cultured in APEL (STEMCELL Technologies) supplemented with 40 ng/ml human stem cell factor (SCF, PeproTech, Rocky Hill, NJ), 20 ng/ml human vascular endothelial growth factor (VEGF, R&D Systems), 20 ng/mL recombinant human bone morphogenetic protein 4 (BMP-4, R&D Systems) and 10 μM Y-27632 (ROCK inhibitor, MedChem Express, Princeton, NJ) to induce hematopoietic specification. On day 6, spin embryoid bodies (EBs) were transferred directly into each well of an uncoated 6-well plate and cultured in NK cell differentiation medium composed of DMEM/F12 (Gibco), 20% EliteGro-Adv (Elitecell), 1% penicillin–streptomycin-solution (Thermo Fisher), 1% GlutaMAX (Thermo Fisher), 1 μM β-mercaptoethanol (Thermo Fisher), 5 ng/mL sodium selenite (Sigma-Aldrich), 50 μM ethanolamine (MP Biomedicals), 20 mg/mL ascorbic acid (Sigma-Aldrich). Cytokines were added as follows: 5 ng/mL IL-3 (PeproTech) (first week only), 20 ng/mL SCF, 20 ng/mL IL-7 (PeproTech), 10 ng/mL IL-15 (PeproTech), and 10 ng/mL Flt3 ligand (PeproTech). Half-media changes were performed weekly. After 28–35 days, differentiated cells expressing CD45⁺CD56⁺CD3⁻ were identified by flow cytometry. These iNK cells were expanded and maintained in a medium of RPMI 1640 supplemented with 10% FBS and 1% penicillin–streptomycin-glutamine (Thermo Fisher), 50 U/mL IL-2 (PeproTech), and 100 ng/mL IL-21 (PeproTech) with media changes every 3–4 days.

**Xenogeneic AML animal models**

To evaluate the anti-tumor efficacy of CD226-overexpression iPSC-derived NK cells (OE226-iNK cells) in vivo, a xenogeneic acute myeloid leukemia (AML) model was established using NOD.Cg-Prkdcscid Il2rgtm1Vst/Vst (NPG) mice. Mice were intravenously injected with 1 × 10⁵ THP1-GL cells (stably expressing firefly luciferase and GFP) to induce systemic leukemia. Mice were randomly administered with IVIS control, Control-iNK, OE226-iNK or left untreated. Twenty-four hours after tumor inoculation, mice were left untreated or injected with 1 × 10⁶ (high dose) Control iNK cells and 3 × 10⁵ (low dose) OE226-iNK cells. All treatments were administered via intravenous injection. To support NK cell survival and function, mice were administered intraperitoneal injections of recombinant human IL-2 (PeproTech) at 500,000 IU/kg every other day (q.o.d.) for 14 days, and recombinant human IL-15 (PeproTech) at 50 μg/kg once daily (q.d.) for 7 consecutive days, starting on the day of iNK cell infusion (day 0).Tumor burden was monitored weekly for up to six weeks using bioluminescence imaging (BLI) with the Xenogen IVIS Spectrum Imaging System (PerkinElmer). All NPG mice were obtained from Beijing Nonc Biotechnology. Animal experiments were conducted in accordance with NIH guidelines and approved by the Institutional Animal Care and Use Committee (IACUC) of Peking University.

**Cytotoxicity assay**

To evaluate the cytotoxic activity of engineered NK cells in vitro, luciferase-tagged tumor cell lines THP1-GL were used as target cells. The cell line were previously established in our laboratory by lentiviral transduction of a firefly luciferase expression vector and maintained under puromycin selection to ensure stable reporter expression. Effector cells including Control and OE226-iNK cells were co-cultured with Luc-expressing target cells in 96-well plates at defined effector-to-target (E:T) ratios. After 24 hours of incubation, cytotoxicity was assessed using a luciferase-based bioluminescence assay as previously described. The percentage of specific lysis was calculated using the following formula: % specific killing = 100 × (spontaneous death RLU − test RLU) / (spontaneous death RLU), where RLU refers to relative luminescence units measured by a microplate reader.

**Flow cytometry**

Cells were stained with antibodies diluted 1:100 in PBS containing 2% FBS for 30 min at room temperature in the dark. Flow cytometry analysis and cell sorting were performed using a Beckman Coulter CytoFLEX and BD FACSAria II, respectively. Data were analyzed with FlowJo software and Prism (GraphPad Software). The antibodies are described in Supplementary Table 1.

**Multiplex cytokines and secreted proteins analysis**

To evaluate cytokine secretion and cytotoxic protein release, we co-cultured iPSC-derived NK cells (Control-iNK and OE226-iNK) with THP1-GL tumor cells at varying E:T ratios. Supernatants were collected from the 1:1 ratio group after 24 hours for analysis. Cytokine and cytotoxic protein levels were quantified using the LEGENDplex™ Human CD8/NK Cell Panel (BioLegend, San Diego, CA) according to the manufacturer’s instructions. The panel enables simultaneous detection of TNF-α, IFN-γ, granzyme A, granzyme B, perforin, and granulysin. Fluorescent bead-based immunoassays were performed and analyzed by flow cytometry, with data processed using LEGENDplex™ Data Analysis Software (BioLegend). Serum levels of IL-6, TNF-α, and IFN-γ were measured using ELISA kits (Thermo Fisher) following the manufacturer’s instructions. Absorbance (450 nm) was read after TMB substrate reaction, and concentrations were calculated from standard curves.

**RNA‑sequencing and data analysis**

Total RNA samples extracted from Control and OE226-iNK cells were purified using a column-based DNase treatment. amples with high purity (A260/280 and A260/230 > 1.7; RIN or RQN > 9) underwent ribosomal RNA depletion prior to library preparation. For transcriptomic profiling, raw sequencing reads were filtered and trimmed using SOAPnuke (v1.5.6), followed by alignment to the human reference genome (GRCh38.p13) using HISAT2 (v2.1.0). Gene expression quantification was performed with RSEM (v1.3.1). Differential expression analysis was conducted using DESeq2 (v1.4.5), with genes defined as differentially expressed if |log2 fold change| ≥ 1 and adjusted P-value (Q-value) ≤ 0.05. Gene Set Enrichment Analysis GSEA software was used to analyze genes function from the MSigDB database, with Q-values used to assess the significance.

**Statistical analysis**

Data are presented as mean ± standard deviation of the mean (SD) for *in vitro* assays with results derived from at least three independent experiments. Group comparisons were performed using unpaired Student's t-test, as appropriate. For *in vivo* imaging analyses and murine inflammatory cytokine measurements, data are shown as mean ± standard error of the mean (SEM), and statistical differences were assessed using unpaired Student’s t-tests. Kaplan–Meier survival curves were analyzed by the log-rank (Mantel–Cox) test. All statistical analyses were conducted using GraphPad Prism software, and a p-value less than 0.05 was considered statistically significant.

**
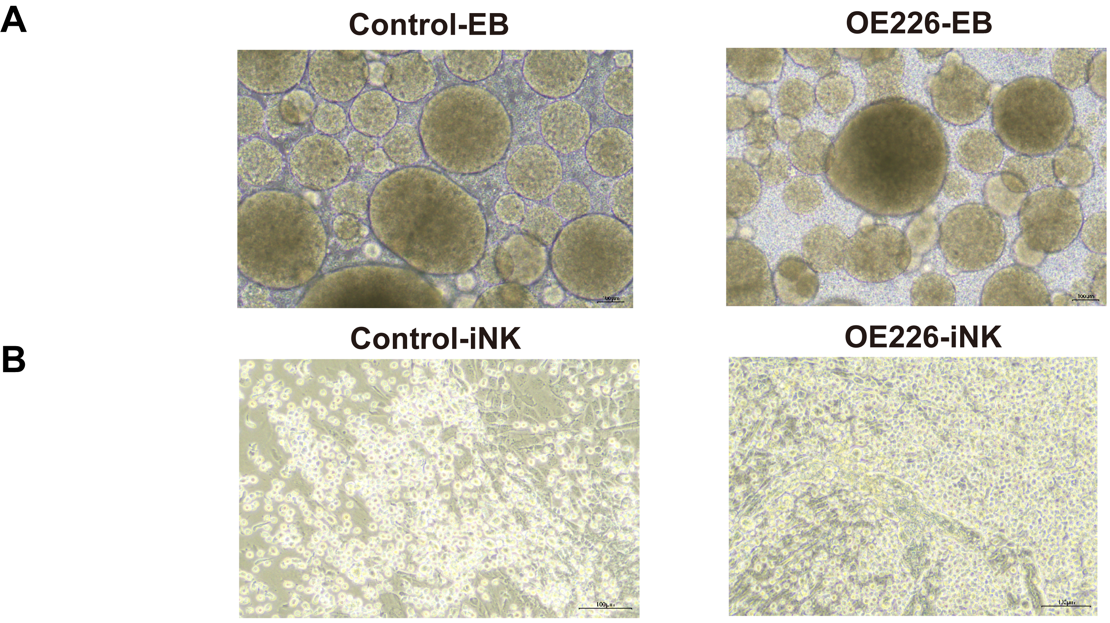
**

**Supplementary Figure 1. NK cell differentiation from Control and OE226-iPSCs. A** Brightfield images of EBs from Control and OE226-iPSCs. Scale bar, 100 μm. **B** Brightfield images of derived iNK cells from Control and OE226-iPSCs. Scale bar, 100 μm.

**
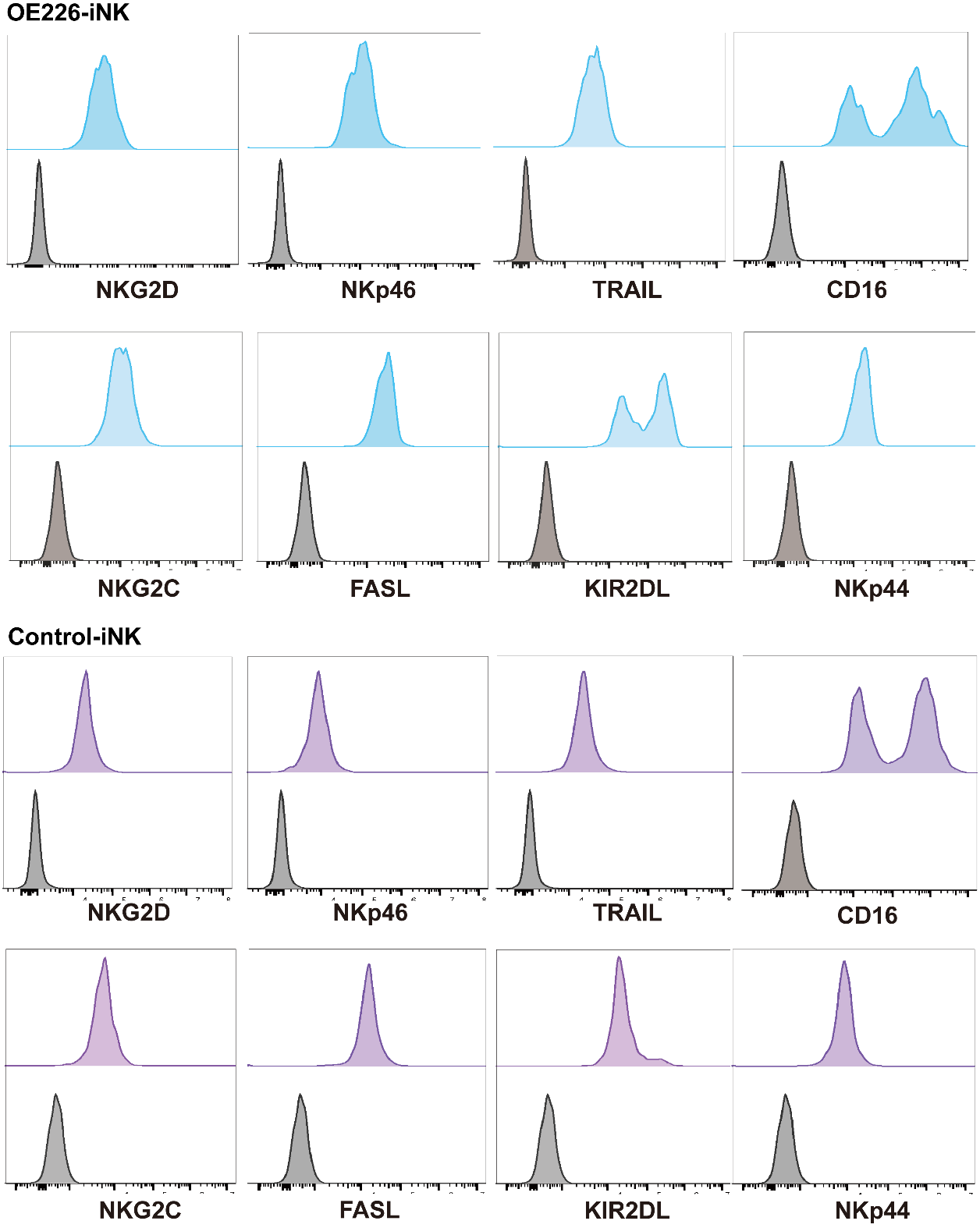
**

**Supplementary Figure 2. Flow cytometry analysis for surface immunophenotyping of OE226-iNK and Control-iNK cells.**

**
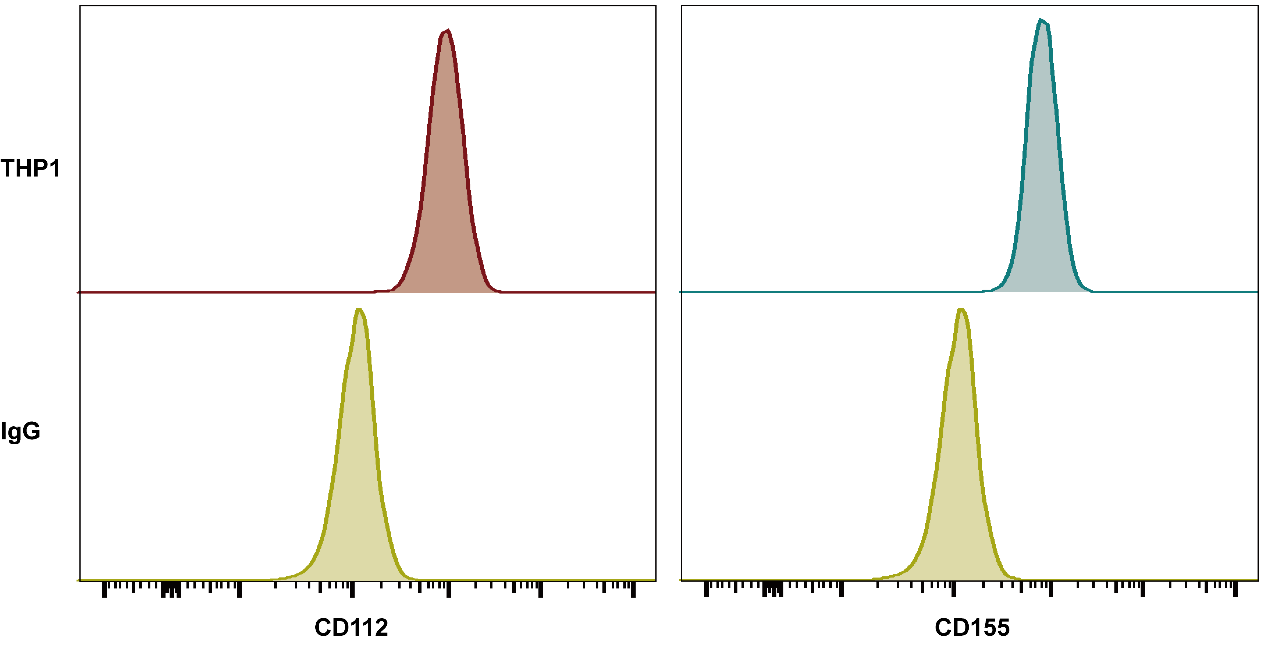
**

**Supplementary Figure 3. Flow cytometry analysis for CD226 ligand expression in THP1-GL cells.**

**Supplementary Table 1. Antibodies used in this study**

| ANTIBODY | SOURCE | CAT | RRID | IDENTIFIFER |
| --- | --- | --- | --- | --- |
| PE anti-human CD34 | BD Biosciences | CAT#555822 | RRID:AB_396151 | CAT#555822 RRID:AB_396151 |
| APC anti-human CD226 | Biolegend | CAT#338312 | RRID:AB_2561951 | CAT#338312 RRID:AB_2561951 |
| PE anti-human 178 (Flt3-L) | Biolegend | CAT#306407 | RRID:AB_2100664 | CAT#306406 RRID:AB_2100664 |
| APC anti-human CD253 (Trail) | Biolegend | CAT#308210 | RRID:AB_2564398 | CAT#308210 RRID:AB_2564398 |
| PE Mouse IgG1, κ isotype Ctrl | Biolegend | CAT#400112 | RRID:AB_2847829 | CAT#400112 RRID:AB_2847829 |
| APC Mouse IgG1, κ Isotype Ctrl (FC) | Biolegend | CAT#400121 | RRID:AB_2665396 | CAT#400121 RRID:AB_2665396 |
| APC anti-human CD159c(NKG2C) Antibody | Biolegend | CAT#375003 | RRID:AB_2888871 | CAT#375003 RRID:AB_2888871 |
| PE anti-human CD45 | Biolegend | CAT#304008 | RRID:AB_314396 | CAT#304008 RRID:AB_314396 |
| PE/Cyanine7 Mouse IgG1, κ isotype Ctrl | Biolegend | CAT#400126 | RRID:AB_326448 | CAT#400126 RRID:AB_326448 |
| APC anti-human CD158 (KIR2DL1/S1/S3/S5) | Biolegend | CAT#339510 | RRID:AB_2565577 | CAT#339510 RRID:AB_2565577 |
| PE/Cyanine7 anti-human CD56 (NCAM) | Biolegend | CAT#318318 | RRID:AB_604107 | CAT#318318 RRID:AB_604107 |
| APC anti-human CD335 (NKp46) | Biolegend | CAT#331918 | RRID:AB_2561650 | CAT#331918 RRID:AB_2561650 |
| PE anti-human CD16 | Biolegend | CAT#302008 | RRID:AB_314208 | CAT#302008 RRID:AB_314208 |
| PE anti-human CD336 (NKp44) | Biolegend | CAT#325108 | RRID:AB_756100 | CAT#325108 RRID:AB_756100 |
| PE anti-human CD314 (NKG2D) | Biolegend | CAT#320806 | RRID:AB_492960 | CAT#320806 RRID:AB_492960 |
| APC anti-human CD56 (NCAM) | Biolegend | CAT#318310 | RRID:AB_604106 | CAT#318310 RRID:AB_604106 |
| APC anti-human CD155 | Biolegend | CAT#337167 | RRID:AB_2565814 | CAT#337167 RRID:AB_2565814 |
| APC anti-human CD112 | Biolegend | CAT#337411 | RRID:AB_2565729 | CAT#337411 RRID:AB_2565729 |

**Abbreviations**

AML: Acute Myeloid Leukemia; iPSC: Induced pluripotent stem cell; NK: Natural Killer Cell; iNK: iPSCs-derived NK; RLU: Relative luminescence unit; FBS: Fetal Bovine Serum; GFP: green fluorescent protein; EB: embryoid body; NPG: NOD. Cg-Prkdcscid Il2rgtm1Vst/Vst; IVIS: In Vivo Imaging System; E:T: Effector-to-target ratio; WT: Wild Type; GSEA: Gene set enrichment analysis; BLI: Bioluminescence imaging; IFN-γ: Interferon-γ; TNF-α: Tumor necrosis factor-alpha; SD: standard deviation of the mean; SEM: standard error of the mean.
